# Adhesion of Resin-Resin and Resin–Lithium Disilicate Ceramic: A Methodological Assessment

**DOI:** 10.3390/ma14143870

**Published:** 2021-07-11

**Authors:** Simon Guggenbühl, Abdulmonem Alshihri, Nadin Al-Haj Husain, Mutlu Özcan

**Affiliations:** 1 Center for Dental and Oral Medicine, Division of Dental Biomaterials, Clinic for Reconstructive Dentistry, University of Zurich, 8032 Zurich, Switzerland; simon.guggenbuehl@gmail.com (S.G.); monem.alshihri@post.harvard.edu (A.A.); nadin.al-haj-husain@zmk.unibe.ch (N.A.-H.H.); 2 Department of Prosthetic Dental Sciences, College of Dentistry, King Saud University, Riyadh 11451, Saudi Arabia; 3 Department of Reconstructive Dentistry and Gerodontology, School of Dental Medicine, University of Bern, 3010 Bern, Switzerland

**Keywords:** adhesion, adhesive, bond strength, ceramic, macroshear, macrotensile, microshear, microtensile, resin composite, surface conditioning, test method

## Abstract

The aim of this study was to evaluate four test methods on the adhesion of resin composite to resin composite, and resin composite to glass ceramic. Resin composite specimens (*N =* 180, Quadrant Universal LC) were obtained and distributed randomly to test the adhesion of resin composite material and to ceramic materials (IPS e.max CAD) using one of the four following tests: (a) Macroshear SBT: (*n =* 30), (b) macrotensile TBT: (*n =* 30), (c) microshear µSBT: (*n =* 30) and (d) microtensile µTBT test (*n =* 6, composite-composite:216 sticks, ceramic-composite:216 sticks). Bonded specimens were stored for 24 h at 23 °C. Bond strength values were measured using a universal testing machine (1 mm/min), and failure types were analysed after debonding. Data were analysed using Univariate and Tukey’s, Bonneferroni post hoc test (α = 0.05). Two-parameter Weibull modulus, scale (m), and shape (_0_) were calculated. Test method and substrate type significantly affected the bond strength results, as well as their interaction term (*p* < 0.05). Resin composite to resin composite adhesion using SBT (24.4 ± 5)^a^, TBT (16.1 ± 4.4)^b^ and µSBT (20.6 ± 7.4)^a,b^ test methods presented significantly lower mean bond values (MPa), compared to µTBT (36.7 ± 8.9)^b^ (*p* < 0.05). When testing adhesion of glass ceramics to resin composite, µSBT (6.6 ± 1)^B^ showed the lowest and µTBT (24.8 ± 7)^C,D^ the highest test values (MPa) (SBT (14.6 ± 5)^A,D^ and TBT (19.9 ± 5)^A,B^) (*p* < 0.05). Resin composite adhesion to ceramic vs. resin composite did show significant difference for the test methods SBT and µTBT (resin composite (24.4 ± 5; 36.7 ± 9 MPa) vs. glass ceramic (14.6 ± 5; 25 ± 7 MPa)) (*p* > 0.05). Among substrate–test combinations, Weibull distribution presented the highest shape values for ceramic–resin in µSBT (7.6) and resin–resin in µSBT (5.7). Cohesive failures in resin–resin bond were most frequently observed in SBT (87%), followed by TBT (50%) and µSBT (50%), while mixed failures occurred mostly in ceramic–resin bonds in the SBT (100%), TBT (90%), and µSBT (90%) test types. According to Weibull modulus, failure types, and bond strength, µTBT tests might be more reliable for testing resin-based composites adhesion to resin, while µSBT might be more suitable for adhesion testing of resin-based composites to ceramic materials.

## 1. Introduction

Adhesive systems and technologies have been dynamic fields during the past decades that presented a wide array of enhanced systems. Currently, the use of a reliable bonding system and meticulous protocol could dictate the retention and long-term success of bonded restorations. Although adhesive technologies have shown major improvements, various factors influence the strength, performance, and longevity of bonded substrates [1,2]. These factors are simultaneously correlated to the bonding zone and to its interaction with bonded substrates of tooth structure or any restorative material. For instance, physiomechanical properties, chemical compositions, surface treatments as well as the characteristics of the adhesive system, are all key factors in outcomes of adhesion [3,4].

The nature of the oral and masticatory system comprises a complex biofunctional environment. The bonded restorative materials are under constant physical, mechanical, and chemical factors on daily basis with various degrees of severity and fluctuation [5]. Thus, to quantify the bonding effectiveness of select materials, preclinical tests must be performed to simulate the physiological conditions to ensure the suitability of materials, surface treatments, and adequate bond strength for successful clinical use. Numerous laboratory tests have been made to simulate the occlusal forces (shear, tensile, compressive, and flexural force) that are exerted on bonded restorations. It is crucial that the applied force targets the adhesive joint between an adherent and a substrate to quantify the bond strength of relevance [5,6]. Such tests, along with other experimental cofactors, provide a general pattern of prediction on how bonded restorations could perform in clinical scenarios. Nonetheless, showing ‘the more realistic’ clinical performance by laboratory tests still falls short of real clinical outcomes [7,8,9].

Numerous studies have shown variations in stress delivery to the adhesive joint when bond strength tests are applied. Shear and tensile bond strength tests (macro- or micro-test) are among the most used static tests to measure bond strength in laboratory settings. In a macro bond test, the force (shear or tensile) is applied to an area of more than 3 mm^2^. These tests however result in heterogeneous stress distribution across the bonding interface, which could skew the obtained outcomes and also could cause fracture patterns within the bulk of tested materials due to uneven stress concentration [10,11,12]. As a result, microshear and microtensile bond strength tests were introduced to overcome the heterogeneously concentrated stress associated with macrotests. Microshear and microtensile tests are utilised with specimens of a small cross-sectional area (<1 mm^2^). In addition, microtests generate more homogeneously distributed stress that could deliver an indicative interfacial adhesion load [6,13,14].

All-ceramic restorations have shown superior aesthetic results and remarkable clinical success. Recent advances in ceramic material types and fabrication techniques have introduced different ceramic options with higher strength and toughness [15], which therefore have made ceramic restorations more applicable in different aspects of dentistry such as posterior teeth restorations and dental implant prosthetics [16,17,18]. It has been shown that using resin luting cement could provide more predictable and durable adhesion to the tooth structure, as well as to ceramic restorations, when compared to conventional luting cement [19,20]. Despite the numerously available ceramic-based and ceramic-like materials, glass-matrix ceramics are conventionally well recognised for their successful and reliable performance with bonding techniques [4,15]. Lithium–disilicate is a glass-matrix ceramic with more than 50% of silica content. In contrast to zirconia ceramics, lithium disilicate is well reactive to ordinary adhesive procedures such as acid etching and silanisation, which maximises its clinical applications and longevity. [4,21] Adhesion between resin cement and glass ceramic is achieved by two main methods, namely, chemical bonding and micromechanical interlocking. Using acid etching and/or airborne particle abrasion micromechanical attachment can be archived [12,22,23], while the chemical bond is primarily established by coupling agents [24]. Moreover, different ceramic types require different bonding protocols due to their inherent variations in chemical compositions and microstructure. Similarly, different bonding agents and protocols can yield different bonding outcomes [14,23,25].

In the oral cavity, multiple forces are applied at different magnitudes on different surfaces of teeth and restorations during mastication. As a single laboratory test cannot simulate nor illustrate the occurring simultaneous forces, it is of clinical value to perform and relate different bond strength tests of bonded complexes and to evaluate the validity of the different testing methods [6,7]. The objective of this study was therefore to investigate the impact of various bond strength testing methods (macro- vs. micro-test) of resin composite adhesion to resin composite and its adhesion to glass ceramic (lithium disilicate). The hypotheses tested were as follows: (1) different test methods would yield different resin bond strength and failure type results for both resin and ceramic and (2) micro-test results would be more consistent and relevant to the adhesive interface of interest, compared to macro-tests.

## 2. Material and Methods

### 2.1. Specimen Preparation

**Figure 1:**
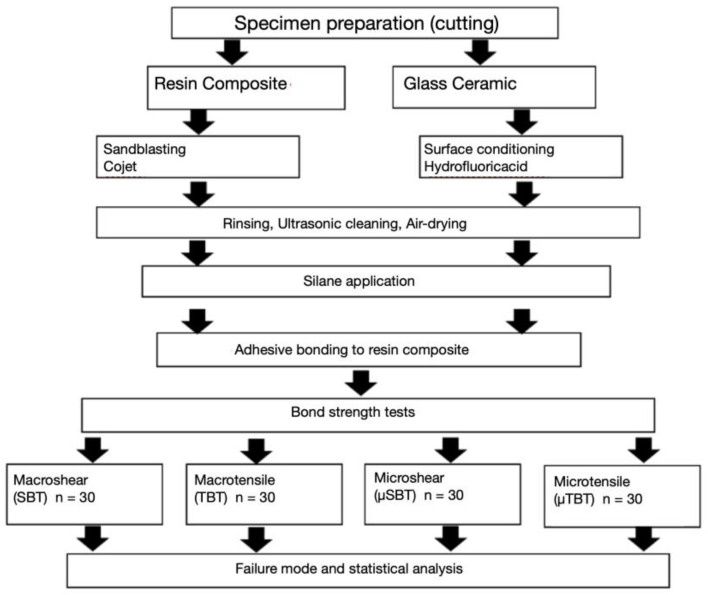
Experimental flowchart presenting distribution of the tested groups according to the testing method, substrate, and procedure sequences of the experiment. SBT: Macroshear, TBT: macrotensile, µSBT: microshear and µTBT: microtensile.

The experimental flowchart explaining the distribution of study groups regarding substrate type, testing method, and experimental procedure sequences is shown in Figure 1.

Material brands, manufacturers, types, and chemical composition of all products used in the study are presented in Table 1. 

The surfaces of the resin composite (Quadrant Universal AC, Shade A3, Caves, Haarlem, The Netherlands) and ceramic (IPS e.max CAD, Ivoclar Vivadent, Vivadent, Schaan, Lichtenstein) were prepared and cut using an electrical precision diamond wire saw (blade diameter 0.17 mm, 30 μm roughness and cutting force) under water cooling (Well, Walther Ebner, Locle, Switzerland). After cutting, they were polished manually under water flow with 1200 grit silicon carbide paper (Streuers, Willich, Germany) until an even flat surface was obtained. The roughness of the surfaces was verified using a digital micrometer (Mitutoyo, Kamagawa, Japan).

Specimens were tested according to the technical specification of ISO/TS 11405 [26].

The resin composite surfaces were air-abraded (CoJet, 30 µm 1.2 bar, 3 M ESPE, St. Paul, MN, USA), with Al_2_O_3_ particles coated with silica with a grain size of 30 µm. Afterwards, they were washed and rinsed. The ceramic surfaces were etched with 5% hydrofluoric acid for 20 s and rinsed for 20 s. Then, all resin composite and ceramic specimens were ultrasonically cleaned in water for 5 min and gently air-dried for 5 s. Finally, a coupling agent (Monobond Plus, Ivoclar Vivadent, Schaan, Liechtenstein) was coated on all specimen surfaces for 1 min and air-dried again.

The number of specimens for each conducted test was as follows: macroshear test (SBT), macrotensile (TBT) and microshear test (µSBT) (*n =* 30; 2.5 × 2.5 mm^2^) each and microtensile test (µTBT) (*n =* 6, composite–composite:216 sticks, ceramic–composite:216 sticks) (3 × 1.5 × 1 mm^3^). Resin composite surfaces (Quadrant Universal AC, Shade A3 Cavex, Haarlem, The Netherlands) were assigned randomly to either resin composite (Quadrant Universal AC, Shade A3 Cavex, Haarlem, The Netherlands) or ceramic (IPS e.max CAD, Ivoclar Vivadent, Schaan, Lichtenstein) substrates.

### 2.2. Bonding

One layer of the bonding agent (Heliobond, Syntac Classic, Ivoclar Vivadent, Schaan, Lichtenstein) was coated using a brush on each specimen for 20 s, air-thinned for 3 s, and photopolymerised for 40 s using an LED polymerisation lamp (Bluephase, Ivoclar Vivadent). For the photopolymerisation a distance of 2 mm was chosen using a light intensity of at least 1200 mW/cm^2^.

Throughout the experiment, one calibrated operator carried out all adhesive procedures. Polytetrafluorethylene (Teflon) moulds with a translucent surface (DuPont, Saint-Gobain, France) were placed on the resin composite or ceramic specimens using a custom-made holder. For each test method, the diameter and height of the moulds were accordingly chosen as follows: for SBT: 4 mm, 2.9 mm; for TBT: 4 mm, 3 mm; for µSBT: 4 mm, 0.8 mm. Moulds were filled using resin composite, and the 100 μm thickness of the first layer of the first increment was ensured using a metal pin prior to photopolymerisation (Bluephase, Ivoclar Vivadent, Schaan, Lichtenstein). Afterwards, the moulds were filled in two increments and polymerised for 40 s from 5 directions. At the bonded margins oxygen inhibiting gel (Oxyguard, Kuraray, Tokyo, Japan) was applied and rinsed after 1 min.

For each specimen, one composite resin block was prepared. The resin composite-composite and composite–ceramic units were bonded using cyanoacrylate adhesive (Super bonder Gel, Loctite Ltd., Sao Paulo, Brazil) upon the metallic base of the cutting machine. For each specimen, the machine calibration was reassessed. The assembly was cut using diamond discs (Accutom50, Stuers A/S, Ballerup, Denmark) under flowing water cooling. Bar-shaped specimens were obtained. External sections were eliminated, and the blocks were fixed on the metallic base after turning 90°. Every composite/ceramic–composite block was used to obtain four transversal sections, from which sticks with a length of 8 mm and an adhesive area of 1 mm^2^ were obtained. Only central external crack-free specimens were evaluated at ×50 magnification under the optical microscope (Zeiss MC 80DX, Jena, Germany). Sticks (*N =* 216) were produced from ceramic and composite groups, and the bonded area was verified using a digital calliper (100 μm) accuracy.

All specimens were kept at room temperature at 23 °C for one day and thereafter subjected to adhesion testing.

### 2.3. Adhesion Tests and Failure-Type Analysis

Specimens of the SBT and µSBT test groups were fixed in the jig of the Universal Testing Machine (Zwick ROELL Z2.5 MA 18-1-3/7, Ulm, Germany). The adhesive interface was loaded close to the substrate until failure using a shearing blade for SBT and µSBT. The stress–strain curve was measured using the software program (TestXpert, testXpert II, 2017, Zwick ROELL, Ulm, Germany). For the TBT test method, the prepared specimens were placed, and the resin composite discs were pulled at a speed of 1 mm/min from the substrate surface using a grip. The sticks obtained for µTBT were fixed with cyanoacrylate glue (Super Bonder Gel, Loctite, IL, USA) to the alignment device on the composite and on the composite/ceramic of the bar. The tensile force was applied until debonding.

Debonded specimens were examined using the optical microscope at ×50 magnification (Zeiss MC 80 DX, Jena, Germany) and the failure types classified in 14 score groups (Score 1–8) as follows: Score 1: cohesive failure in the substrate; Score 2a: mixed cohesive failure in substrate and cohesive or adhesive failure in bond (>50% in bonding); Score 2b: mixed cohesive failure in substrate and cohesive or adhesive failure in bonding (> or <50% in bonding and 50% in substrate); Score 2c: mixed cohesive failure in substrate and cohesive or adhesive failure in bonding (>50% in substrate); Score 3: adhesive failure between substrate and bonding; Score 4a: mixed cohesive and adhesive failure in bonding (>50%) adhesive failure between bonding and substrate; Score 4b: mixed cohesive and adhesive failure in bonding (>50%) cohesive failure in bonding; Score 4c: mixed cohesive and adhesive failure in bonding (>50%) adhesive failure between bonding and adherent; Score 5: adhesive failure between bonding and adherent; Score 6a: mixed cohesive failure in adherent and cohesive or adhesive failure in bonding (>50% in Bond); Score 6b: mixed cohesive failure in adherent and cohesive or adhesive failure in bonding (> or <50% in bonding and 50% in adherent; Score 6c: mixed cohesive failure in adherent and cohesive or adhesive failure in bonding >50% in adherent); Score 7: cohesive failure in adherent; Score 8: cohesive failure in substrate, bonding, and adherent.

### 2.4. Statistical Analysis

Statistical analyses of bonded specimens were performed using the Statistical Package for Social Sciences (version 18.0, 2011, SPSS Inc, Chicago, IL, USA). Shapiro–Wilk and Kolmogorov–Smirnov tests showed the normal distribution of the data. Univariate analysis of variance was used to analyse differences between the groups. The dependent variables were the bond strength, substrate type (resin composite vs. ceramic), and the testing methods (SBT; TBT; µSBT; µTBT). Interactions were analysed using Tukey’s or Bonneferroni post hoc tests. Two-parameter Weibull distribution (Minitab Software V.16, State College, PA, USA) was used to evaluate reliability and predictability of the adhesion, while two-sided chi-square testing was used for failure type analysis. A *p* value less than 0.05 was considered significant.

## 3. Results

Both substrate type (*p* < 0.05) and test method (*p* < 0.05) significantly affected the bond strength and their interactions (*p* < 0.05).

**Figure 2:**
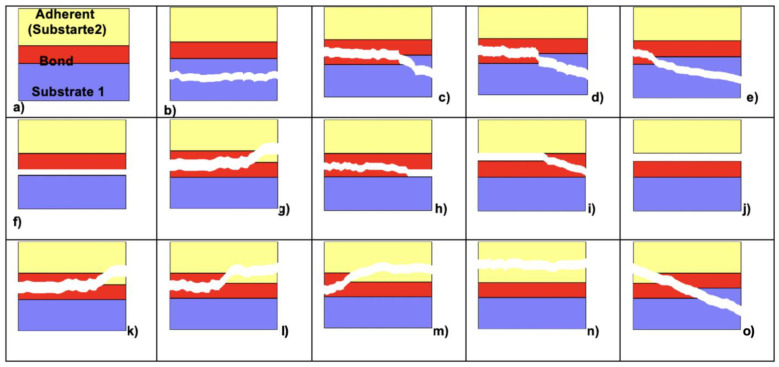
(**a**–**o**) Schematic sketch of 14 different evaluated failure types: (**a**) specimen before treatment, showing 3 different sections, upper layer is the adherent (substrate 2), the lowest one is the substrate 1, and both are bonded through a bond layer; (**b**) cohesive failure in substrate; (**c**) mixed cohesive failure in substrate and cohesive or adhesive failure in bond (>50% in bond); (**d**) mixed cohesive failure in substrate and cohesive or adhesive failure in bond (> or <50% in bond and 50% in substrate); (**e**) mixed cohesive failure in substrate and cohesive or adhesive failure in bond (>50% in substrate); (**f**) adhesive failure between substrate and bond; (**g**) mixed cohesive and adhesive failure in bond (>50%) adhesive failure between bond and substrate; (**h**) mixed cohesive and adhesive failure in bond (>50%) cohesive failure in bond; (**i**) mixed cohesive and adhesive failure in bond (>50%) adhesive failure between bond and adherent; (**j**) adhesive failure between bond and adherent; (**k**) mixed cohesive failure in adherent and cohesive or adhesive failure in bond (>50% in bond); (**l**) mixed cohesive failure in adherent and cohesive or adhesive failure in bond (> or <50% in bond and 50% in adherent; (**m**) mixed cohesive failure in adherent and cohesive or adhesive failure in bond >50% in adherent); (**n**) cohesive failure in adherent; (**o**) cohesive failure in substrate, bond, and adherent.

When testing adhesion of resin composite to resin composite, SBT (24.4 ± 5)^a^, TBT (16.1 ± 4.4)^b^ and µSBT (20.6 ± 7.4)^a,b^ test methods showed significantly lower mean bond values (MPa), compared to µTBT (36.7 ± 8.9)^b^ (*p* < 0.05), (Figure 2).

When testing adhesion of glass ceramics to resin composite, µSBT (6.6 ± 1 MPa)^B^ showed the lowest and µTBT (24.8 ± 7 MPa)^C,D^ the highest test values, while SBT (14.6 ± 5 MPa)^A,D^ and TBT (19.9 ± 5 MPa)^A,B^ presented values in between (*p* < 0.05).

**Figure 3:**
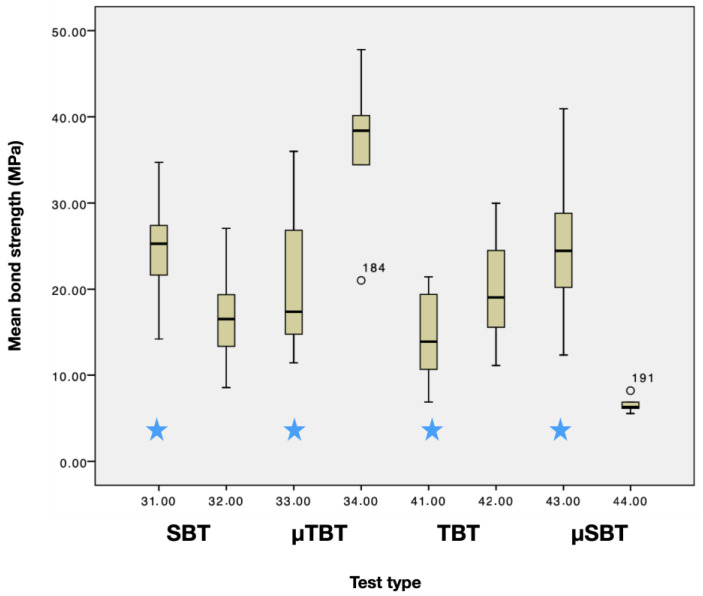
Boxplot of mean bond strength values for different test methods of composite–composite (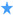) and ceramic–composite assemblies.

Adhesion of resin composite to glass ceramic vs. resin composite did show a significant difference for the test methods SBT and µTBT (resin composite (24.4 ± 5 MPa; 36.7 ± 9 MPa) vs. glass ceramic (14.6 ± 5 MPa; 25 ± 7 MPa)) (*p* > 0.05) Boxplots are shown in (Figure 3). The Weibull distribution showed the highest shape values for ceramic–resin µSBT (7.6) and resin–resin µSBT (5.7) among all substrate–test combinations.

**Figure 4:**
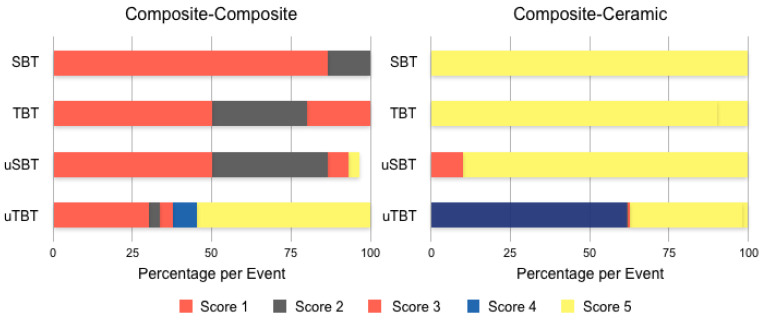
Distribution of failure modes for composite–composite (**left**) and ceramic–composite assemblies (**right**).

To simplify the statistical analysis of the failure types, they were reduced to five subgroups, Subscore 1: cohesive1: cohesive failure in the substrate (Score 1,8); Subscore 2: mixed1: a combination of adhesive and cohesive failure types in the substrate and bonding agent (Score 2a–c); Subscore 3: adhesive: adhesive failure of bonding agent from the resin composite surface with no remnants on the resin composite (Score 3,4a–c,5); Subsore 4: mixed2: a combination of adhesive and cohesive failure types in the bonding agent and resin composite (Score 6a–c); Subscore 5: cohesive2: cohesive failure in the resin composite (Score 7). Figure 4 and Table 2 and Table 3 show the detailed distribution and frequency of all 14 possible failure types occurring in all experimental groups.

Cohesive failures in resin–resin bond were the most frequently observed failure types in SBT (87%), TBT (50%), and µSBT (50%), while mixed cohesive adhesive failures occurred most in ceramic–resin bonds in the test types SBT (100%), TBT (90%), and µSBT (90%) (Table 4, Table 5 and Table 6).

## 4. Discussion

This study was conducted to evaluate the adhesion of resin composite to glass ceramic (lithium disilicate) and to resin composite. Four adhesion tests (macroshear, microshear, macrotensile, and microtensile) were used to measure the bond strength of resin composite to composite and to ceramic. The mode of failure was also assessed to correlate bond strength to the quality of bonding.

Although clinical studies are the ultimate evidence of material performance, laboratory tests go hand in hand with clinical studies to single out possible factors of shortcomings. The main difference between macro- and micro-tests is the area of adhesion, and consequently, the interfacial stress distribution. Regardless of what bond strength test is used, the interface bond should uniformly be the stress-receiving zone. When quantifying the bond strength, it is essential to examine the quality of tested adhesive joints (adhesive vs. cohesion fracture). Macro-tests have been criticised based on the fact that they induce non-uniform stress at the interfacial area [10,27]. Additionally, the inherent material characteristics of substrates are a major factor in bond strength outcomes. Test methods and substrate types significantly affected the bond strength values in this study. Other studies showed similar outcomes with various substrates [28]. The first hypothesis of this study is thus accepted.

The adhesion of resin composite to ceramic vs. resin composite showed a significant difference for the test methods SBT and µTBT. These findings are consistent with one other study (SBT) in which different substrate materials showed a significant effect on the SBS [29]. µTBT showed the most indicative outcomes for composite adhesion to both composite and ceramic, and associated failure mode. These findings are in line with the outcomes of numerous studies that showed the consistency and reliability of µTBT, compared to TBT, as well as other tests. It was reported in a meta-analysis that µTBT was the most commonly used test, within the search criteria, and seemed to have a larger discriminative power, compared to macrotests [30]. Although this review looked at the bonding to dentin, which could be interpreted differently from our tested materials, it is still relevant to clinical predictability and related tests. Moreover, testing outcomes can be influenced by a number of factors involved in the experimental setup. Numerous studies have pointed out possible variations in the outcomes and nonuniform distribution of stress for most of the tests that are currently used, shear, microshear, tensile [26,31], and microtensile [32].

Although µTBT has been considered a reliable test to examine bond strength irrespective of the tested material, it can also be influenced by other factors such as specimen shape/geometry (stick, dumbbell, hourglass), flaws during specimen preparation/adhesion, the thickness of the bonding agent, the angle of loading, and modulus of elasticity differences among the tested materials [33,34].

Bonding of glass ceramics to resin composite showed for µSBT (6.6 ± 1 MPa)^B^ the lowest and µTBT (24.8 ± 7 MPa)^C,D^ the highest test values (SBT (14.6 ± 5 MPa)^A,D^ and in between TBT (19.9 ± 5 MPa)^A,B^) (*p* < 0.05). Shear bond strength, when alternating adherent and substrate of glass ceramic and composite, was shown to be affected by the type of adherent-substrates assembly [29] and by the size of bonded area. Previous studies have demonstrated that the bonded area and bond strength are adversely correlated, which is not the case in our study, where SBT is higher than microSBT for both [29]. As regards ceramic–composite failure mode for SBT, TBT, and µSBT, 90–100% of the specimens for these tests showed a mixed fracture pattern, namely, a combination of adhesive and cohesive failure in the bonding interface and resin composite. µTBT of ceramic–composite assembly showed mainly (61%) cohesive failure in the substrate. As for the composite–composite, SBT, TBT, and µSBT failure mode analyses presented mainly (50–80%) a cohesive failure in the substrate, whereas µTBT showed a cohesive failure in the adherent in 54.6% of the specimens, and 30% of the specimens showed a cohesive failure of in the substrate. The difference of elastic moduli between an adherent and substrates can contribute to the dissimilar results in bond strength. Different mechanical performance (SBS) was shown with mismatched elastic moduli of adherent and substrates [29]. Additionally, localised stress concentration at the bonding interface can result from the mismatch of the elastic moduli of adherent and substrates [35,36].

Performing different bond strength tests is expected to result in a separation of bonded bodies, which therefore quantify the bond strength. Ideally, this separation should occur at the adhesive joint to reflect its performance accurately. Otherwise, results of cohesive patterns of fracture can be erroneous findings of an adhesive system. External load shrinkage strain can be redirected towards the substrate when bonded to an adherent with a higher elastic modulus. On the other hand, adherents with low elastic modulus are less likely to redistributed stains and rather show tensile cracking as a result of restrained shrinkage [29,34].

The quality of adhesion is also influenced by the bonding mechanisms. Specific surface treatments are used to maximise the micromechanical or chemical retention of different or similar materials [23,37]. The micromechanical retentive preparation of ceramic surfaces plays a vital role in bonding with resin. Morphology modification of the ceramic surface was shown in numerous studies utilising different approaches such as hydrofluoric acid (HFA) and airborne particle abrasion. In this study, Hydrofluoric acid was used to condition the ceramic surface for its superior retention performance, as shown in multiple studies [38,39,40]. For composite surface preparations, different studies showed that air abrasion and resurfacing with silane can be very effective to bond to resin composite [41,42,43,44,45].

Future clinical studies should verify the outcomes of this study and confirm the validity of the methods for testing adhesion of resin-based materials to resin-based composites or to ceramic materials. Nevertheless, the effect of the adhesion test method should be considered when ranking the adhesion performance of resin composites to different substrate materials.

## 5. Conclusions

The following could be concluded from this study:

1. The resin composite to resin composite adhesion showed significantly higher values with µTBT. SBT values were significantly higher than that of TBT but not significantly different from µSBT.
2. The resin composite adhesion to lithium disilicate glass ceramic was significantly higher with µTBT and SBT, and the lowest with TBT and µSBT.
3. Only with SBT and µTBT, a significant difference could be observed for bond values between resin–resin and resin–ceramic combinations.
4. Using µTBT, Weibull distribution indicated more reliable adhesion of resin composite to resin composite and ceramic.
5. Except for µTBT, cohesive failure in the substrate was more frequent in resin–resin combinations, compared to resin–ceramic combinations. Similarly, except for µSBT, adhesive failure was more frequent in resin–resin combinations, compared to resin–ceramic combinations.
6. Mixed failures occurred mostly in resin–ceramic adhesion with SBT (100%), TBT (90%), and µSBT (90%) test types.

**Clinical Relevance:** Adhesion of resin composite to ceramic materials and other resin composite materials should be tested In vitro using different test methods. µTBT tests could be considered more suitable for testing the adhesion of resin-based materials to resin-based composites, while µSBT could be more suitable for testing the adhesion of resin-based materials to ceramics. Considering bond strength values and failure types, the adhesion of resin composite to itself is more reliable than to ceramic and can be considered more reliable clinically.

## Figures and Tables

**Table 1:** Product names, manufacturers, and the respective chemical compositions of study material.

Product Name	Manufacturer	Chemical Composition
IPS e.max CAD	Ivoclar Vivadent AG, Schaan, Lichtenstein	>57% SiO_2_, Li_2_O, K_2_O, P_2_O_5_, ZrO_2_, ZnO, Al_2_O_3_, MgO, pigments
IPS ceramic etching gel	Ivoclar Vivadent AG, Schaan, Lichtenstein	5% hydrofluoric acid, water
Monobond Plus	Ivoclar Vivadent AG, Schaan, Lichtenstein	Silane methacrylate, phosphoric acid methacrylate, sulphide methacrylate, ethanol
Heliobond	Ivoclar Vivadent AG, Schaan, Lichtenstein	bis-GMA (50–100), Triethylenglycoldimethacrylate (25–50%)
Qadrant Universal LC	Cavex, Haarlem, The Netherlands	Feldspar 20–25%; Bisphenol A Diglycidyl Methacrylate (bis-GMA) 10–20%, Silica (0.1–2.5%)

**Table 2:** Mean bond strength values (MPa ± standard deviations) of all 4 test methods (SBT; TBT; µSBT; µTBT), distribution, and frequency of failure types, Weibull modulus. Failure types were classified as follows: Score 1: cohesive1: cohesive failure in the substrate; Score 2: mixed1: a combination of adhesive and cohesive failure types in the substrate and bonding agent; Score 3: adhesive: adhesive failure of bonding agent from the resin composite surface with no remnants on the resin composite; Score 4: mixed2: a combination of adhesive and cohesive failure types in the bonding agent and resin composite; Score 5: cohesive2: cohesive failure in the resin composite. The same superscript lowercase letters in the same column indicate no significant differences based on the substrate type, and uppercase letters are based on the test method (*p* < 0.05). Test group descriptions are explained in Figure 1. Significantly different bond strengths in comp-comp substrates are marked with the superscripts a, b, c, while significantly different values for Ceramic-comp substrates were distinguished using the superscripts A, B, C and D.

						Weibull Modulus (m) (95% CI)			Failure Type Distribution n (%)				
Group	Substrate	Test Method	Produced/Pre-Test Failures/Final Analyzed Specimens	Bond Strength (Mean ± SD)	Min-Max (95% CI)	Shape	Scale	CI Shape	Score 1 (1+8)	Score 2 (2a–c)	Score 3 (3,4a–c,5)	Score 4 (6a–c)	Score 5 (7)
1	Comp–Comp	SBT	30/0/30	24.4 ± 5.0 ^a^	14.2–34.7 (22.5–26.3)	5.50	26.4	(4.19–7.21)	26 (86.7%)	4 (13.3%)	0 (0%)	0 (0%)	0 (0%)
2	Comp–Comp	TBT	30/0/30	16.1 ± 4.4 ^b^	8.6–27.1 (14.4–17.7)	4.07	17.7	(3.10–5.34)	15 (50%)	9 (30.0%)	6 (20%)	0 (0%)	0 (0%)
3	Comp–Comp	µSBT	30/0/30	20.6 ± 7.4 ^a,b^	11.4–36.0 (17.9–23.4)	3.07	23.12	(2.33–4.03)	15 (50%)	12 (36.6%)	0 (0)	2 (6.6%)	1 (3.3%)
4	Comp–Comp	µTBT	216/0/216	36.7 ± 8.9 ^c^	21.0–47.8 (27.4–46.0)	5.68	39.78	(2.96–10.93)	65 (30.1)	8 (3.7%)	9 (4.1%)	16 (7.4%)	118 (54.6%)
5	Ceramic–Comp	SBT	30/0/30	14.6 ± 4.8 ^A,D^	6.9–21.4 (12.8–16.4)	3.51	16.3	(2.62–4.69)	0 (0%)	0 (0%)	0 (0%)	30 (100%)	0 (0%)
6	Ceramic–Comp	TBT	30/0/30	19.9 ± 5.3 ^A,B^	11.1–30.0 (17.9–21.8)	4.24	21.88	(3.21–5.61)	0 (0%)	0 (0%)	0 (0%)	27 (90%)	3 (10%)
7	Ceramic–Comp	µSBT	30/0/30	6.6 ± 0.9 ^B^	12.3–40.9 (22.1–27.6)	3.75	27.51	(2.86–4.94)	0 (0%)	0 (0%)	3 (10%)	27 (90%)	0 (0%)
8	Ceramic–Comp	µTBT	216/0/216	24.8 ± 7.4 ^C,D^	5.6–8.2 (5.6–7.5)	7.64	6.95	(4.30–13.6)	133 (61.6)	1 (0.5%)	1 (0.5%)	77 (35.7%)	4 (1.9%)

**Table 3:** Frequency and distribution of failure type per experimental group analysed after bond strength test: Score 1: cohesive failure in the substrate; Score 2a: mixed cohesive failure in substrate and cohesive or adhesive failure in bond (>50% in bond); Score 2b: mixed cohesive failure in substrate and cohesive or adhesive failure in bond (> or <50% in bond and 50% in substrate); Score 2c: mixed cohesive failure in substrate and cohesive or adhesive failure in bond (>50% in substrate); Score 3: adhesive failure between substrate and bond, Score 4a: mixed cohesive and adhesive failure in bond (>50%) adhesive failure between bond and substrate; Score 4b: mixed cohesive and adhesive failure in bond (>50%) cohesive failure in bond; Score 4c: mixed cohesive and adhesive failure in bond (>50%) adhesive failure between bond and adherent; Score 5: adhesive failure between bond and adherent; Score 6a: mixed cohesive failure in adherent and cohesive or adhesive failure in bond (>50% in bond); Score 6b: mixed cohesive failure in adherent and cohesive or adhesive failure in bond (> or <50% in bond and 50% in adherent; Score 6c: mixed cohesive failure in adherent and cohesive or adhesive failure in bond >50% in adherent); Score 7: cohesive failure in adherent, Score 8: cohesive failure in substrate, bond, and adherent.

		Failure Type Distribution n (%)													
Group	Substrate	Score 1 (1)	Score 2 (2a)	Score 3 (2b)	Score 4 (2c)	Score 5 (3)	Score 6 (4a)	Score 7 (4b)	Score 8 (4c)	Score 9 (5)	Score 10 (6a)	Score 11 (6b)	Score 12 (6c)	Score 13 (7)	Score 14 (8)
1	Comp-Comp	26 (86.7%)	4 (13.3%)	0 (0%)	0 (0%)	0 (0%)	0 (0%)	0 (0%)	0 (0%)	0 (0%)	0 (0%)	0 (0%)	0 (0%)	0 (0%)	0 (0%)
2	Comp-Comp	12 (40.0%)	0 (0%)	0 (0%)	9 (30.0%)	0 (0%)	6 (20.0%)	0 (0%)	0 (0%)	0 (0%)	0 (0%)	0 (0%)	0 (0%)	0 (0%)	3 (10.0%)
3	Comp-Comp	13 (43.3%)	1 (3.3%)	0 (0%)	11 (33.3%)	0 (0%)	0 (0%)	0 (0%)	0 (0%)	0 (0%)	0 (0%)	1 (3.3%)	1 (3.3%)	1 (3.3%)	2 (6.67%)
4	Comp-Comp	61 (28.2%)	7 (3.2%)	1 (0.5%)	0 (0%)	0 (0%)	0 (0%)	9 (4.1%)	0 (0%)	0 (0%)	6 (2.8%)	3 (1.4%)	7 (3.2%)	118 (54.6%)	4 (1.9%)
5	Ceramic-Comp	0 (0%)	0 (0%)	0 (0%)	0 (0%)	0 (0%)	0 (0%)	0 (0%)	0 (0%)	0 (0%)	30 (100%)	0 (0%)	0 (0%)	0 (0%)	0 (0%)
6	Ceramic-Comp	0 (0%)	0 (0%)	0 (0%)	0 (0%)	0 (0%)	0 (0%)	0 (0%)	0 (0%)	0 (0%)	0 (0%)	1 (3.3%)	26 (86.7%)	3 (10%)	0 (0%)
7	Ceramic-Comp	0 (0%)	0 (0%)	0 (0%)	0 (0%)	3 (10%)	0 (0%)	0 (0%)	0 (0%)	0 (0%)	18 (60%)	3 (10%)	6 (20%)	0 (0%)	0 (0%)
8	Ceramic-Comp	0 (0%)	1 (0.5%)	0 (0%)	0 (0%)	0 (0%)	0 (0%)	1 (0.5%)	0 (0%)	0 (0%)	0 (0%)	1 (0.5%)	76 (35.2%)	4 (1.9%)	133 (61.6%)

**Table 4:** Significant differences of bond strength values of resin composite to resin composite on the test method. For group descriptions see Figure 1.

Comp-Comp	SBT	TBT
SBT	-	0.000
TBT	0.000	-
µSBT	0.515	0.269
µTBT	0.004	0.000

**Table 5:** Significant differences of bond strength values of ceramic to resin composite on the test method. For group descriptions see Figure 1.

Ceramic–Comp	SBT	TBT
SBT	-	0.111
TBT	0.111	-
µSBT	0.000	0.156
µTBT	0.227	0.001

**Table 6:** Cross-comparison of significant differences between mean bond strengths of composite-to-composite versus ceramic-to-resin composite based on the test method.

Comp–Comp vs. Ceramic–Comp	SBT	TBT	µSBT	µTBT
SBT	0.000	0.266	1.000	0.000
TBT	0.996	0.518	0.000	0.074
µSBT	0.035	1.000	0.354	0.000
µTBT	0.000	0.000	0.006	0.000

## Data Availability

All the data is available within the manuscript.
